# Supplementary material for: Loan maturity aggregation in interbank lending networks obscures mesoscale structure and economic functions
Source: Sci Rep. 2019 Aug 29;9:12512. doi: 10.1038/s41598-019-48924-5 (PMC6715684; doi:10.1038/s41598-019-48924-5)
Supplement: Supplementary file 1 — Loan maturity aggregation in interbank lending networks obscures mesoscale structure and economic functions (supplementary material) [file 41598_2019_48924_MOESM1_ESM.pdf]

Loan maturity aggregation in interbank lending networks obscures mesoscale structure and economic functions (supplementary material)

Marnix Van Soom[1], Milan van den Heuvel[2,3,*], Jan Ryckebusch[2], and Koen Schoors[3]

[1]Vrije Universiteit Brussel, Artificial Intelligence Lab, Brussels, 1050, Belgium

[2]Ghent University, Department of Physics and Astronomy, Ghent, 9000, Belgium

[3]Ghent University, Department of Economics, Ghent, 9000, Belgium

[*]Milan.vandenHeuvel@UGent.be

Contents

A Maturity layer analysis 2

B OG inference with non-contiguous binning 18

C Number of active banks per group 19

D Activity characteristics per OGB 20

E Other percentiles for instrength/strength of important banks 21

F The trust crisis 22

G Maturity classes in the ILN literature 24

H Term structure of interest rates and loan volumes in the interbank loan market 25

References 28

A Maturity layer analysis

Here we give a detailed description and network analysis of the Russian interbank lending network to compare to other interbank networks used in research. We divide the Russian interbank loan network $\{G_\alpha\}$ by maturity class α into separate layers consisting only of the loans with loan maturity inside each maturity class. (Note that in the main text we use the symbol l instead of α.) We evaluate the properties per maturity layer (often also referred to as layer for simplicity) to show the differences between them. We denote the eight maturity layers as: $G_{<1d}$, G_{2-7d}, G_{8-30d}, G_{31-90d}, $G_{91-180d}$, $G_{0.5-1y}$, G_{1-3y}, $G_{>3y}$.

Activity and volume

One expects that the number of banks participating in a given loan maturity layer decreases as the loan maturity lengthens. Table 1 shows that this is approximately correct. More importantly, one sees that the number of active banks becomes very small for G_{1-3y} and $G_{>3y}$. We define an active bank as a bank that has lent or borrowed at least once in a given time window and maturity layer. We will always only consider active banks to deal with the death and birth of banks in the Russian interbank market over the course of time[1]. For the layers with long loan maturity, this results in an exceedingly high average lending activity per bank, comparing to layers with shorter loan maturity. It seems that $G_{>3y}$ is occupied by a small club of banks that trade relatively intense.

Table 1. (top panel) The number of active banks involved in loans by maturity layer and year. A total of 1,040 banks are present in the data. (lower panel) The number of loans per active bank by maturity layer and year, obtained by dividing the top panel of Table 2 and the top panel of this Table element-wise. Care must be taken when interpreting; this is not indicative of an 'average' bank since the degree distributions are heavy-tailed (as will be discussed below). Also note that the columns '1998' and '2004' are biased because they are incomplete in the data (respectively 5 and 10 months missing months).

	1998	1999	2000	2001	2002	2003	2004
<1d	610	810	878	954	1,022	1,027	983
2-7d	589	907	953	1,026	1,074	1,098	1,058
8-30d	375	849	920	980	1,018	1,054	986
31-90d	166	535	648	698	706	775	686
91-180d	63	203	265	310	321	429	378
0.5-1y	49	120	132	169	204	258	259
1-3y	24	64	80	60	58	113	107
>3y	11	45	29	38	33	23	20

	1998	1999	2000	2001	2002	2003	2004
<1d	13	118	187	258	286	337	225
2-7d	7	57	108	161	183	229	147
8-30d	3	15	32	43	44	59	43
31-90d	1	5	10	11	15	18	12
91-180d	1	2	3	4	5	7	5
0.5-1y	1	2	2	4	4	7	7
1-3y	2	3	3	3	3	8	8
>3y	5	3	6	10	78	74	44

Table 2 lists the yearly, and average monthly lending activities by loan maturity class, where we define the *lending activity*

simply as the number of loans recorded during a certain period. A crucial observation is that the lending activity sharply decreases for longer maturities, which is also reported for other interbank markets in[2]. We see that the overnight segment ($G_{<1d}$) is the most active, together with G_{2-7d}. The impacts of the crises in 1998 and 2004 have had clear impact on the lending activity, except for the longer maturities; these seem relatively unaffected.

Table 2. (top panel) Lending activity, defined as number of of loans, by loan maturity class and year. Note that 1998 and 2004 are incomplete years in the data, counting 5 and 10 months respectively. (lower panel) Monthly average lending activity by loan maturity layer. Time series of the lending activity can be found in Fig. 1 in the main text.

	1998	1999	2000	2001	2002	2003	2004
<1d	7,917	95,389	163,855	245,840	292,048	346,221	220,979
2-7d	4,325	51,673	103,376	165,602	196,765	251,006	155,834
8-30d	958	12,914	29,250	42,067	45,204	62,190	42,181
31-90d	245	2,770	6,365	7,939	10,606	14,184	8,443
91-180d	82	460	742	1,189	1,754	2,797	2,010
0.5-1y	63	237	307	593	886	1,901	1,899
1-3y	52	207	279	202	180	914	845
>3y	55	128	180	367	2,588	1,712	890

	1998	1999	2000	2001	2002	2003	2004
<1d	1,583	7,949	13,655	20,487	26,550	28,852	22,098
2-7d	865	4,306	8,615	13,800	17,888	20,917	15,583
8-30d	192	1,076	2,438	3,506	4,109	5,182	4,218
31-90d	49	231	530	662	964	1,182	844
91-180d	16	38	62	99	159	233	201
0.5-1y	13	20	26	49	81	158	190
1-3y	10	17	23	17	16	76	84
>3y	11	12	15	31	235	143	89

Table 3 lists the total loan volumes (i.e. loan sizes) by loan maturity layer and year. We observe that the relative importance of each maturity segment, as measured by the total volume of loans traded within it, follows the ranking of the loan maturity lengths. The loan volumes are log-normally distributed[3], especially for shorter loan maturities.

Table 3. Total loan volumes by loan maturity and per year in billions of rubles.

	1998	1999	2000	2001	2002	2003	2004
<1d	226.3	2,674.4	5,984.3	8,855.9	10,306.4	13,844.6	13,040.1
2-7d	83.1	1,030.4	2,910	5,114.7	6,104.6	9,058.1	7,880.2
8-30d	15.9	220.7	620.8	1,103.1	1,102.9	1,871.8	1,833.3
31-90d	4.4	31.9	105.2	205.3	318.3	415.4	253.6
91-180d	1.7	5.3	9.6	23.4	46.9	73.6	73.7
0.5-1y	3.2	2.4	8	20.2	24	50.8	69.9
1-3y	2.2	5.5	6	6	3.3	10.1	11
>3y	2.6	0.8	1.2	1.4	1.3	0.8	2.5

Maturity layer activity of banks

A node i is defined as active on layer α if it has at least one connection on this layer, i.e. its degree $k_i^\alpha > 0$. In symbols,

$$b_i^\alpha = \begin{cases} 1 & \text{if } k_i^\alpha > 0, \\ 0 & \text{otherwise.} \end{cases} \quad (1)$$

The *total activity* B_i measures the number of layers the node participates in, i.e. $B_i = \sum_\alpha b_i^\alpha$[4]. Figure 1 shows the distribution of B_i on monthly time scales. We see that the average total activity grows steadily as the network develops, finally settling around at a value of about three. Almost no banks make use of more than six maturity layers on a monthly basis. The distribution of the total activity is quite broad and relatively unpeaked, especially during the stable phase of the network. This has been reported as a typical quality of real-world multiplex network [5].

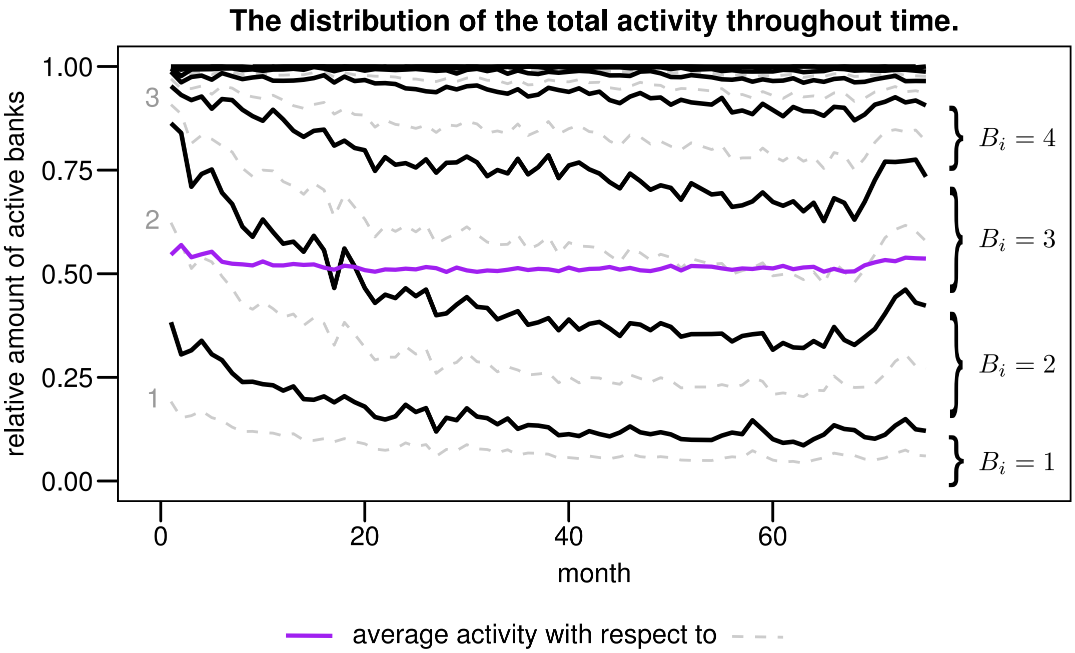

Figure 1. For a given month, the area between the thick lines is proportional to the relative number of active banks with a certain B_i value, which is indicated on the right. The dashed lines make up the moving coordinate system of $\langle B_i \rangle$, in relation to which the average total activity indicated by the thick purple line must be understood. For example, $\langle B_i \rangle$ is about 2 (3) for month 1 (75). The summer of 2004 crisis is mirrored by the drop in $\langle B_i \rangle$ during the last months.

Density

Real-world interbank networks are typically *sparse*, meaning that in directed and undirected views of the network only a small fraction of all possible edges exist. One can then ask how the edges are distributed among the banks. To this end, we define the *degree* of a node. For undirected networks, this is the number of edges connected with a given node, i.e. the number of the bank's counterparties. For directed networks, the in-degree (out-degree) of a node is the number of incoming (outgoing) edges. The in-degree (out-degree) of a bank is then simply the number of loans borrowed (lent) by it.

The above seen differences in lending and bank activity per loan maturity layer is reflected in the directed density of the layers, shown in Fig. 2. All layers can be considered sparse, except for G_{1-3y} and $G_{>3y}$, which have respectively moderate and an extremely high density[6]. We also observe that the density in almost all layers grows steadily as the network develops, pointing to an increasing interconnectedness with increasing development of the market[7].

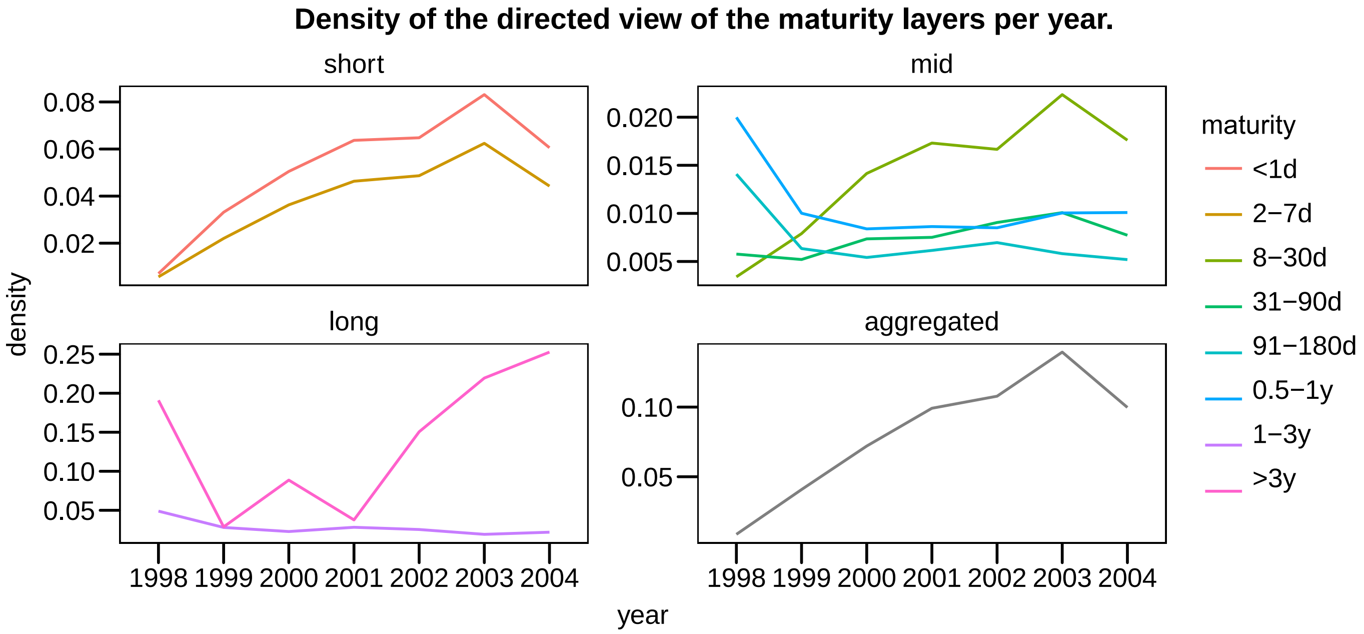

Figure 2. The yearly density per loan maturity layer in the directed Russian interbank lending network. The directed density for a simple graph with N nodes and E edges is defined as $d = \frac{E}{N(N-1)}$.

Degree distribution

Interbank networks, like many real-world networks[8], exhibit heavy-tailed degree distributions. In a nutshell, this means that *few nodes have many links and many nodes have few links*. In general, many typical distributions are heavy-tailed – in fact, they abound in descriptions of natural events like avalanches, earthquakes, turbulent flow and rainfall[9,10]; another example in economics are the non-Gaussian return distributions in financial markets.

Early work in the previous decade tended to postulate power laws for the degree distributions. Networks whose degree distributions follow a power law (at least asymptotically) are called *scale-free* networks. However, while agreeing on the heavy-tail character, recent literature has cast serious doubt on the idea that power laws are the best candidate for the degree

distributions, and thus on the scale-free character of interbank networks. In our case, power laws have been decisively rejected as best fit candidates for the heavy-tailed degree distributions of the <1d and 2-7d loan maturity by Vandermarliere et al. (2015)[3].

We plot the heavy-tailed degree distributions with complementary cumulative distribution functions (ccdfs)[10] with doubly logarithmic scales. If we denote the degree and its distribution by k and $p(k)$ respectively, the ccdf is given by

$$\mathrm{ccdf}(k) \equiv 1 - \mathrm{cdf}(k) = P\left(k \leq k'\right) = \int_k^\infty p(k')\,\mathrm{d}k', \tag{2}$$

where $\mathrm{cdf}(k)$ is the usual cumulative distribution function.

The degree distributions are shown per loan maturity layer in Figures 3 and 4. Each loan maturity layer exhibits fat-tailed in- and out-degree distributions, with the maximum degrees separated some 5 ($G_{>3y}$, in-degree) to 18 (G_{8-30d}, in-degree) standard deviations from the mean. Vandermarliere et al. (2015)[3] established that stretched exponentials of the form

$$f(d) = Cd^{\beta-1}\exp\left(-\lambda d\right)^{\beta} \tag{3}$$

provide the best overall fit for the bulk+tail multi-directed in- and out-degree for monthly and yearly time windows. In Eq. 3, d is the in-degree or out-degree, C a normalization constant and λ, β distribution parameters. The stretched exponential can be understood as a Weibull distribution, with β being the shape parameter, and $1/\lambda$ the scale parameter. If $0 < \beta < 1$, the distribution has a fat tail, with a smaller β putting more weight towards smaller d. $1/\lambda$ widens the distribution, the mean of f being proportional to it.

We fitted f to the degree distributions to see if the result by Vandermarliere et al. (2015)[3] can be extended to longer maturities. The conclusions are identical for the in- and out-degrees. First, the stretched exponential fit does seem to describe the time-aggregated degree distributions of $G_{<1d}$, G_{2-7d} and G_{8-30d}. Then the leap in residual sum-of-squares suggests that G_{31-90d} and longer maturities can not be satisfactorily described by the stretched exponential. This is confirmed by visual inspection. While (parts of) the bulk distributions of the longer loan maturities are reasonably well captured by f, the fits systematically underestimate the ccdf in the tail; put differently, they underestimate for a given large degree d how many nodes exists with an even larger degree d'.

To investigate whether frequent lenders are also frequent borrowers, we first rank the banks with regard to in- and out-degree, then we calculate Kendall's coefficient of concordance W, which measures the rate of agreement $0 < W < 1$ between two rankings, per loan maturity layer. The significant results together with the joint degree distributions are shown in Fig. 5. We conclude that lending and borrowing are highly correlated for $G_{<1d}$, G_{2-7d}, and G_{8-30d}; in other words, the dominant lenders are likely to be dominant borrowers, and vice versa. This correspondence breaks down markedly for the layers with longer loan maturity, with orderly decreasing W but insignificant test results. This can also be seen in the shapes of the contour plots in Fig. 5: high in-degree - out-degree correlation is marked by squeezed contours around the plot diagonal, which does not hold

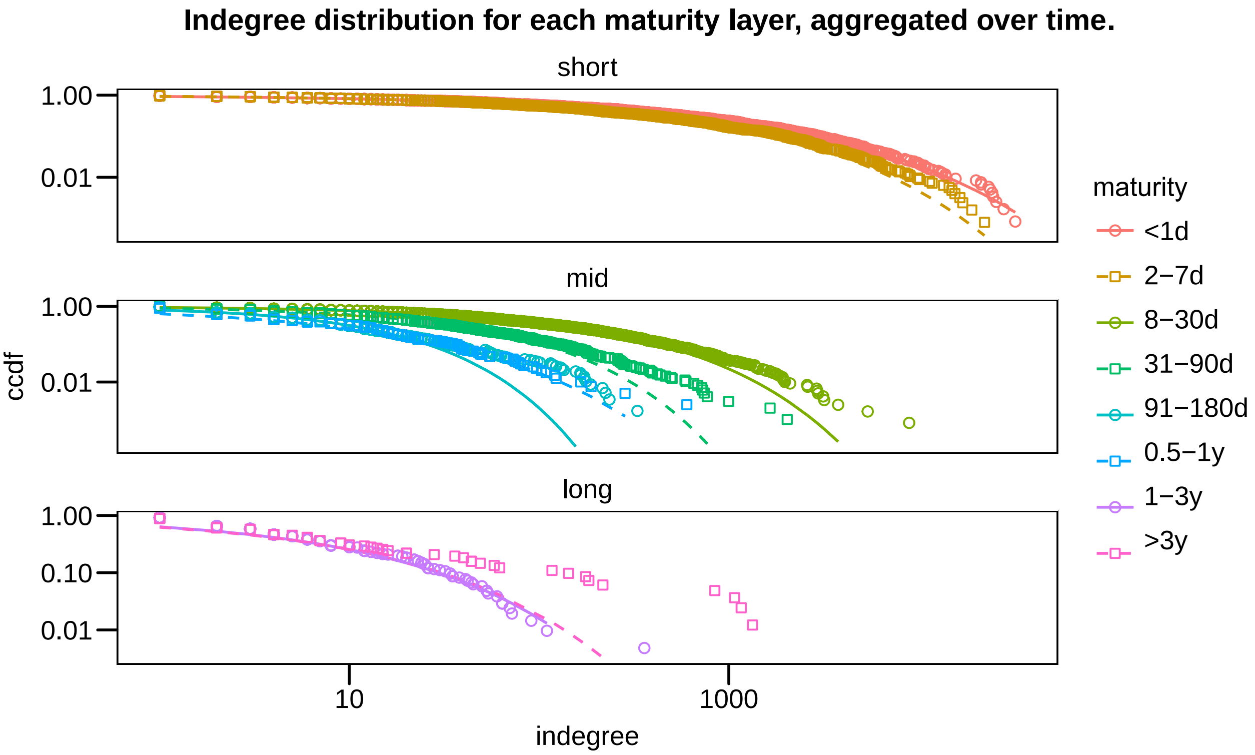

Figure 3. In-degree distribution with attempts to fit the bulk+tail with stretched exponentials, drawn as piecewise linear functions connecting each predicted value. The correspondence with this distribution seems to break down for layers with loan maturity longer than 8-30d. Inferred (λ, β) parameters for $G_{<1d}$, G_{2-7d}, G_{8-30d} are $(2.6e-3, 4.3e-1)$, $(3.4e-3, 4.8e-1)$, $(1.0e-2, 5.8e-1)$ respectively, with all standard errors below 1%.

for maturity longer than 31-90d. G_{1-3y} and $G_{>3y}$ even display modest anti-correlation for the degree tails, meaning that the most frequent lenders (borrowers) area unlikely to be among the most frequent borrowers (lenders).

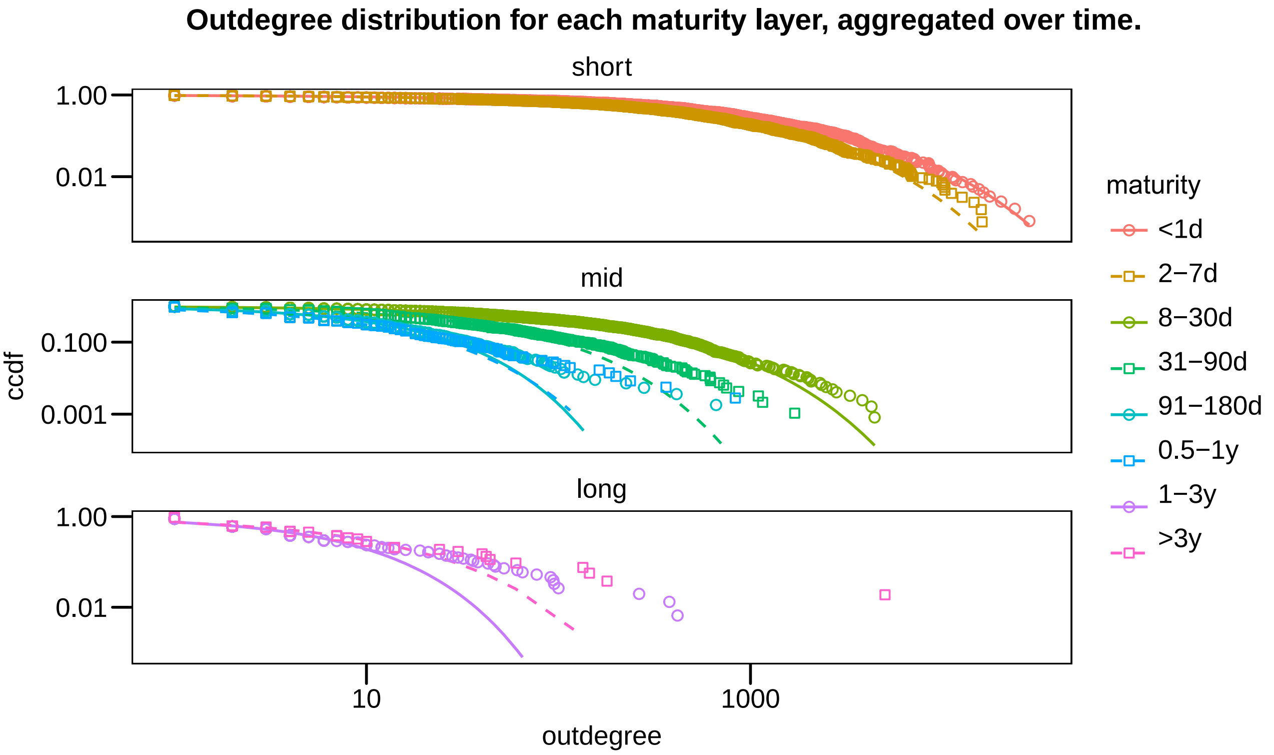

Figure 4. Out-degree distribution with with attempts to fit the bulk+tail with stretched exponentials, drawn as piecewise linear functions connecting each predicted value. It is clear the correspondence with this distribution breaks down for layers with loan maturity longer than 8-30d. Inferred (λ,β) parameters for $G_{<1d}$, G_{2-7d}, G_{8-30d} are $(1.5e-3, 5.3e-1)$, $(2.3e-3, 5.7e-1)$, $(8.3e-3, 6.1e-1)$ respectively, with all standard errors below 1%.

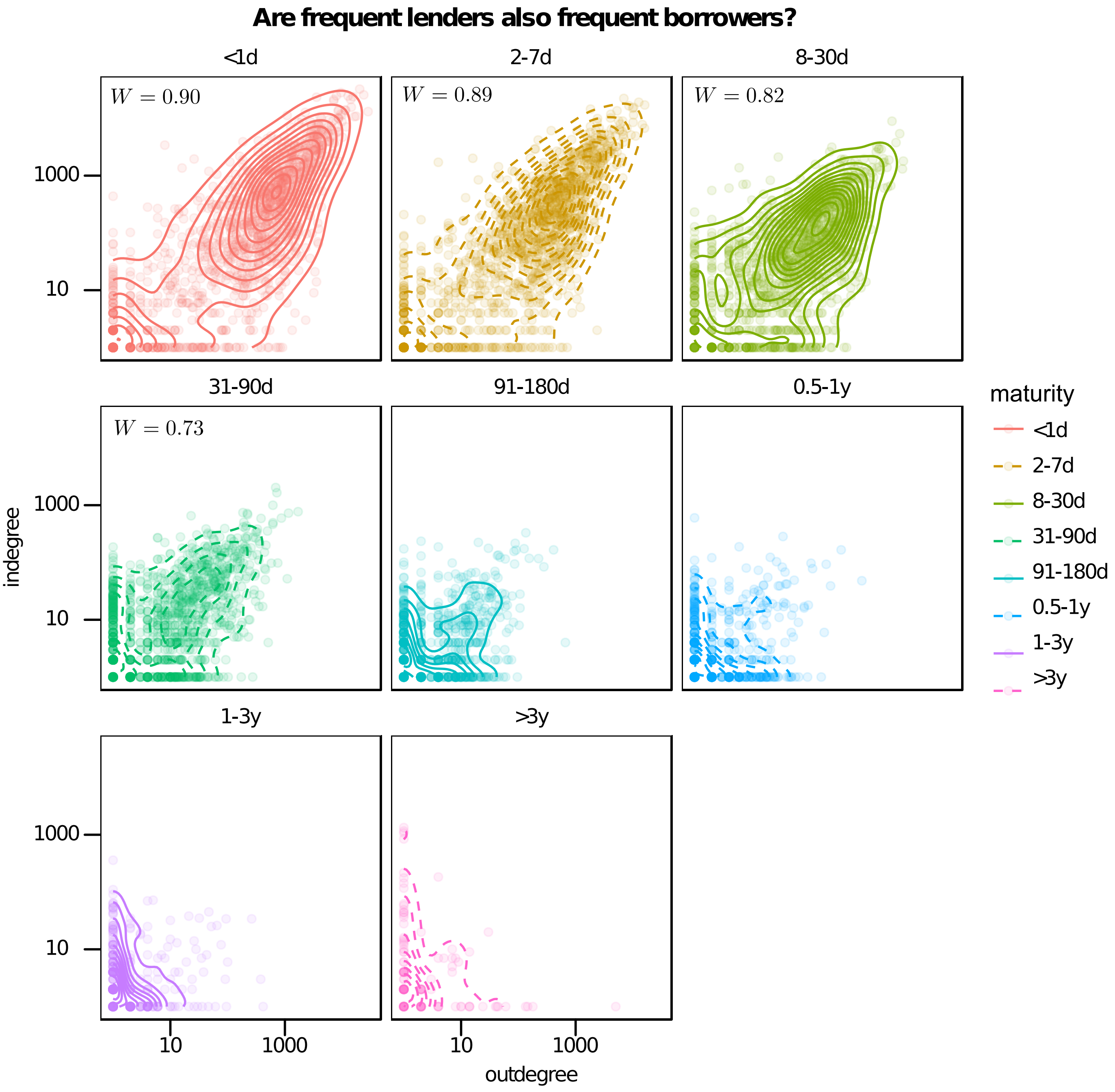

Figure 5. The time-aggregated joint degree distribution for each layer, drawn as density contour plots with almost transparent data points. For the first four maturity bins, a highly significant Kendall W was obtained and put in the top left corner of the panel. Note that all scales are equal for easy comparison.

Average shortest path length

The average shortest path length (denoted from here on as just 'average path length') D is defined for the largest connected component of the undirected view of interbank networks. It indicates the typical distance between two randomly chosen nodes, i.e. the smallest number of edges needed to reach one node from the other. *Small-world networks* are characterized by high clustering and small average path length. Both should be compared to their mean values in an ensemble of random networks with the same number of nodes and edge [11].

Most studies find that D is small for interbank networks, which indicates compact network structure, but not all conclude that they are small-worlds. Hubs tend to lower the average path length; scale-free networks are ultra-small-worlds. D is a measure of the typical length of intermediation chains that are taking place among the market participants (at least in the largest connected component). Longer intermediation chains arise when D is large, which effectively contribute to slowing down the market transactions between participants and consequently harming the liquidity allocation between financial institutions. In contrast, when D is small, the information between the market participants flows quickly in the network, giving rise to a well-functioning liquidity allocation in the market[12].

In the case of the Russian interbank network, the average path length peaks at times when the network is in crisis, and decreases gradually when the network is maturing. The same patterns have been reported in other empirical literature, such as in[13].

Figure 6 shows that exclusively for the layers with loan maturity <90d the average path length D *decreases* roughly linearly when the size of the largest connected component (LCC) increases *logarithmically*. Herein the LCC covers the vast majority of the active banks within yearly time windows, see Fig. 7. Thus we see that core-periphery structure, which we expect in these layers from our analysis in the Topology section, is extremely effective in channeling and intermediating liquidity; of course this comes at the cost of systemic risk in the core hubs.

The remaining layers display the familiar pattern: the size of the largest connected component shrinks for longer maturities, while simultaneously the network becomes increasingly fragmented as the number of unconnected components increase, relative to the number of active banks (Fig. 8) – both tend to suppress D.

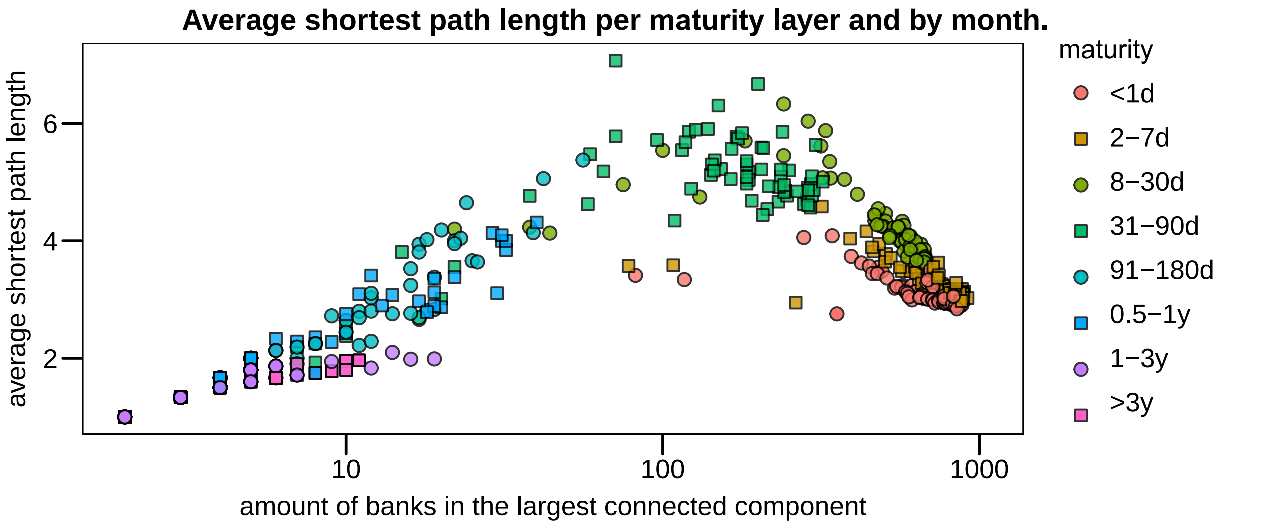

Figure 6. The average (shortest) path in function of the size of the largest (weakly) connected component. For the four shortest loan maturity layers, the points lying astray from the dense cloud all occur during the first five months. Note that the number of banks in the largest connected component for these layers is notably larger than those of the remaining layers.

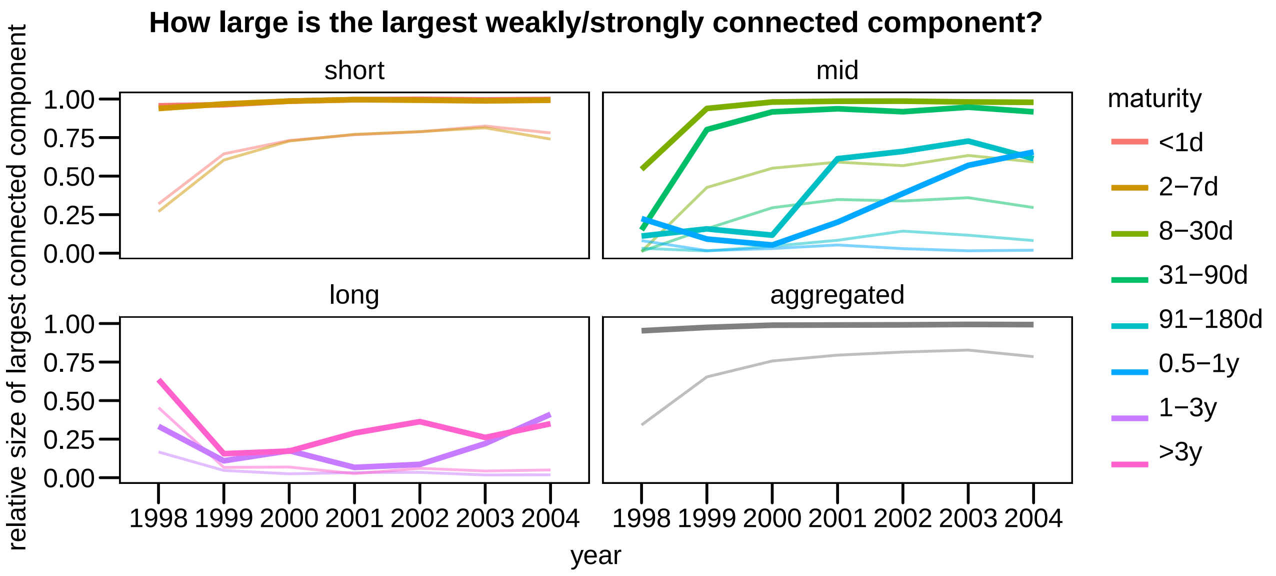

Figure 7. The relative size of the largest connected component, given the weak or strong requirement, is calculated by dividing the number of banks in the largest connected component by the total number of active banks in a specific layer and year. The fat (thin) lines indicate the weak (strong) version.

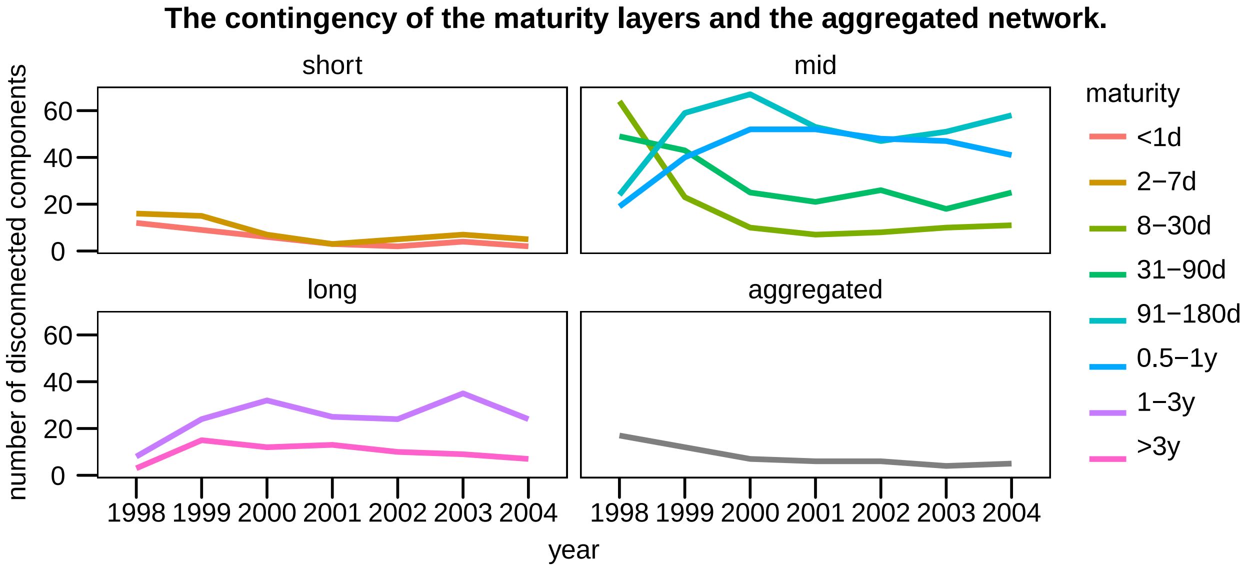

Figure 8. To compare the number of (weakly) disconnected components with the number of active banks per year, consult Table 1 on page 2. The relative size of the largest weakly connected can be found in Fig. 7.

Clustering coefficient

Clustering coefficients measure to which extent banks form triangles. Put differently, they measure the tendency of connected nodes to have common neighbors in undirected views of interbank networks.

According to Bargigli et al.[2], an inverse relationship between the degree and the clustering of a node is observed quite commonly. In the core-periphery picture, low clustering values of core nodes indicate that they essentially behave as star centers. A star graph exhibits zero clustering, as the periphery nodes are unconnected amongst themselves. Consequently, deviations from the star graph, which has idealized core-periphery structure, can be probed by measuring the local clustering coefficients of the periphery nodes. One can identify these heuristically by simply considering the nodes with low degree, so the aforementioned inverse relationship hints that the core-periphery structure may exhibit considerable complexity. A second and more robust implication is that the clustering coefficient of the complete interbank network is dominated by the clustering of low-degree nodes if this inverse relationship is observed together with heavy-tailed degree distributions.

High clustering has obvious implications for systemic risk[3] and therefore clustering coefficients are of interest to the interbank network literature. Table 1 in the main text shows, that at least a few studies claim opposite 'typical' values for clustering coefficients. This may be due to the fact that the clustering tends to increase with longer time windows[2], or that the coefficients may not have been compared to the mean clustering coefficient obtained for random networks of the same size and number of edges.

The *local* clustering coefficient[8] of a node i is defined in function of triangle motifs in undirected networks:

$$C_i = \frac{\text{number of of triangles connected to } i}{\text{number of triples on } i}, \tag{4}$$

where a triple on i means an unordered pair of nodes connected via i, and possibly connected directly. If that is the case, the triple is counted as a triangle, ending up in the nominator of Eq. 4 as well. Thus C_i expresses the degree of connectedness among the neighbors of i[14]. The (*global*) clustering coefficient of an undirected (N,E) network is then simply the average of the local coefficients:

$$C = \frac{1}{N}\sum_i C_i. \tag{5}$$

To assess whether an observed network possesses a non-random clustering structure and thus a significant clustering coefficient C_0, we generate n random networks of the same size, i.e. the same number of nodes and edges, having clustering coefficients $C_{\text{rand}} = \{C_1, C_2, \ldots, C_n\}$. Then we can calculate the z-score of the observed C_0 as

$$z = \frac{C_0 - \langle C_{\text{rand}} \rangle}{\text{sd}(C_{\text{rand}})}. \tag{6}$$

Large z indicates a significant value of C_0, the sign indicating more $(+)$ or less $(-)$ clustering than a random network of the

same size.

We have calculated clustering coefficients and z-scores for the undirected view of the loan maturity layers using yearly time windows which are displayed in Fig. 9. A clear pattern can be seen: the clustering varies from significantly high to moderate (in order $G_{<1d}$, G_{2-7d}, and G_{8-30d}), via moderately low to insignificant (in order G_{31-90d}, $G_{91-180d}$, and $G_{0.5-1y}$), to significantly low (G_{1-3y} and $G_{>3y}$). Since all layers exhibit heavy-tailed degree distributions, the sum in Eq. 5 is dominated by the (local) clustering coefficients of banks with small degree. We can use C to proxy the clustering of the periphery banks

Within that approximation, one sees that the core-periphery structure expected in the short maturity layers deviates considerably from the idealized star network, at least when looked at with yearly resolution. Notwithstanding the strong presence of intermediation found earlier, the lower-tier banks still trade extensively with each other, indicating that contagion risk is not located entirely in the high-tier banks.

In contrast, the two longest maturity layers, where source-sink structures are expected, exhibit considerable star-like structure, low degree nodes and periphery banks being equivalent in most cases. In addition to the low clustering, we recall the modest anticorrelation between in- and outdegree; these observations lead us to believe that G_{1-3y} and $G_{>3y}$ behave as a sort of *source-sink star layers*. These layers have star-like hub structure, but hardly any intermediation occurs in the hubs. They act simply as sources and sinks, generating and dissipating excess liquidity in the interbank network. Of course, one could argue that for loans with maturities of at least one year, no intermediation is possible within the scope of one year. Looking at Fig. 5, which completely aggregates time, however, we see that the banks with the largest degrees tend to be *either* sinks or sources, especially in $G_{>3y}$. Thus the tendency of the hubs to be either sink or source, but not an intermediary, holds for time scales longer than one year. The next question is whether the star centers in the source-sink star layers are connected, or rather at the center of disconnected components. Disconnected components are plentiful compared to the number of active banks per year (see Fig. 8 and Table 1), and less than half of the latter participate in the largest (weakly) connected component, as Fig. 7 shows. Furthermore, we report that in the two source-sink star layers the average shortest path length is between 2 and 3 (see Fig. 6, which shows the average path length for G_{1-3y} and $G_{>3y}$ on a *monthly* basis as being typically 2), which, together with the proven existence of hubs, indicates a compact star-like structure. In a nutshell, the source-sink star layers G_{1-3y} and $G_{>3y}$ are composed in general of many disconnected 'islands', of which the largest exhibit an almost perfect star-like structure around a sink or source hub.

All results are highly significant, except for $G_{91-180d}$ and $G_{0.5-1y}$: these layers do not possess any structural clustering structure on their own. As always, the clustering in the aggregated network resembles mainly the first two layers. We further note that clustering is most volatile in times of crisis, and increases during the growing phase of the network[3].

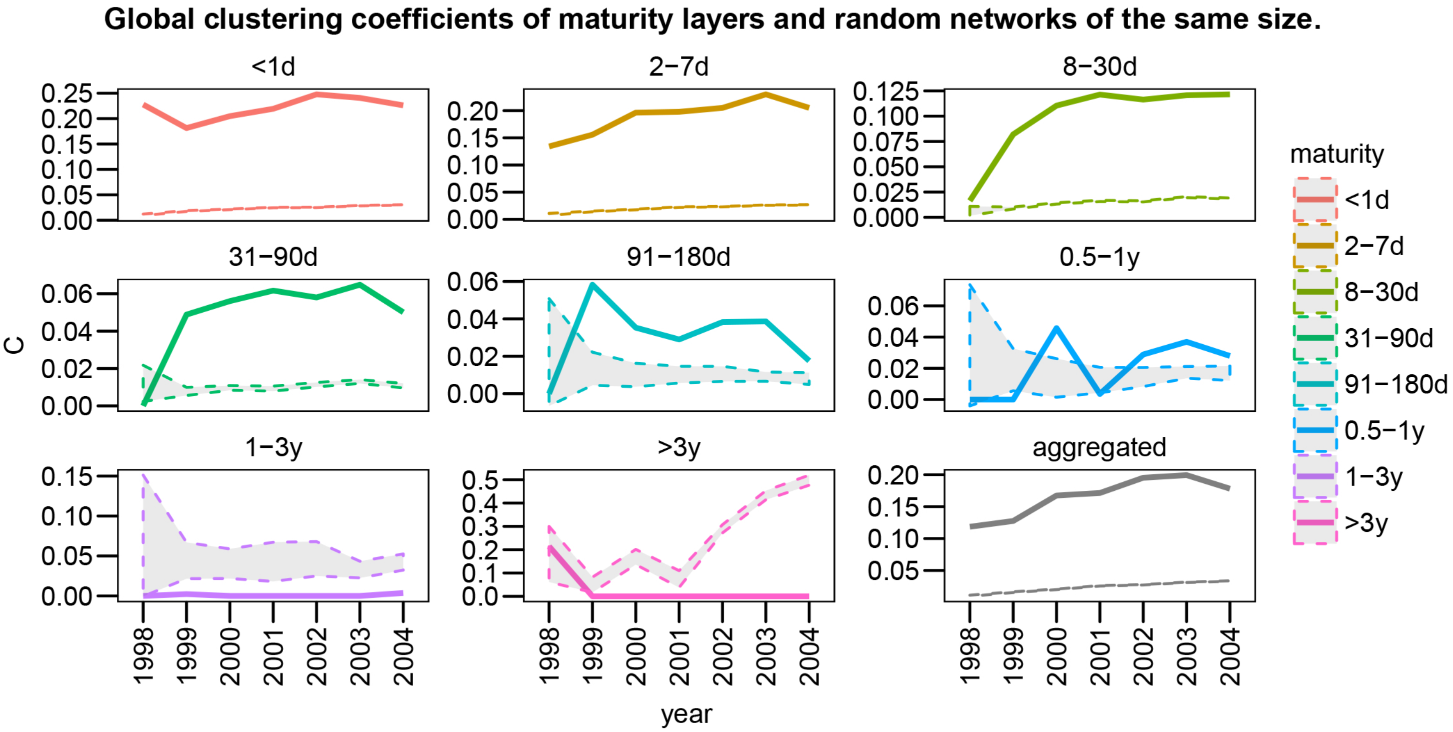

Figure 9. Time series of clustering coefficients for the loan maturity layers and the aggregated network, together with a ribbon centered around $\langle C_{\text{rand}} \rangle$ of total width $2 \times \text{sd}(C_{\text{rand}})$. Following the methodology in[2], a total of 100 Erdős–Rényi networks were generated to test the significance of the observed C's.

Bank size mixing as degree size mixing

Many studies point to *disassortative mixing* with respect to the bank size, meaning that small banks trade mainly with large banks and vice versa. Given the core-periphery picture in these studies, one expects that the core banks are the large banks. Indeed, it is found that total bank assets are significant in explaining core membership[15]. As bank size correlates with total degree, we would also expect disassortative mixing of the banks with respect to the (total) degrees, i.e. high (low) degree nodes tend to be connected to low (high) degree nodes[8]. In fact several studies report this as an additional stylized fact[2,16].

Figure 10 shows the assortativity with respect to the total node degrees on yearly basis, which is also called the total degree correlation. The layers with short maturity show a clear preference for the low-degree nodes to attach to the high-degree ones. This preference weakens when we look at longer maturity layers. The assortativity or the long maturity layers is pushed up by the large number of disconnected clusters, in most cases simply isolated pairs of trading banks. The disassortative degree mixing in the larger clusters is present, caused by their star-like structure. As the number of unconnected components grows drastically, we would expect that the same mechanism is behind the high assortativity for $G_{91-180d}$ and especially for $G_{0.5-1y}$. Interestingly, this expectation turns out to be false: evaluating the degree assortativity only in the largest connected component results in lower values for all layers (notably in those where star-like structures were expected based on the clustering coefficient), but it remains strongly positive for $G_{91-180d}$ and $G_{0.5-1y}$ during the stable phase of the network (Fig. 11).

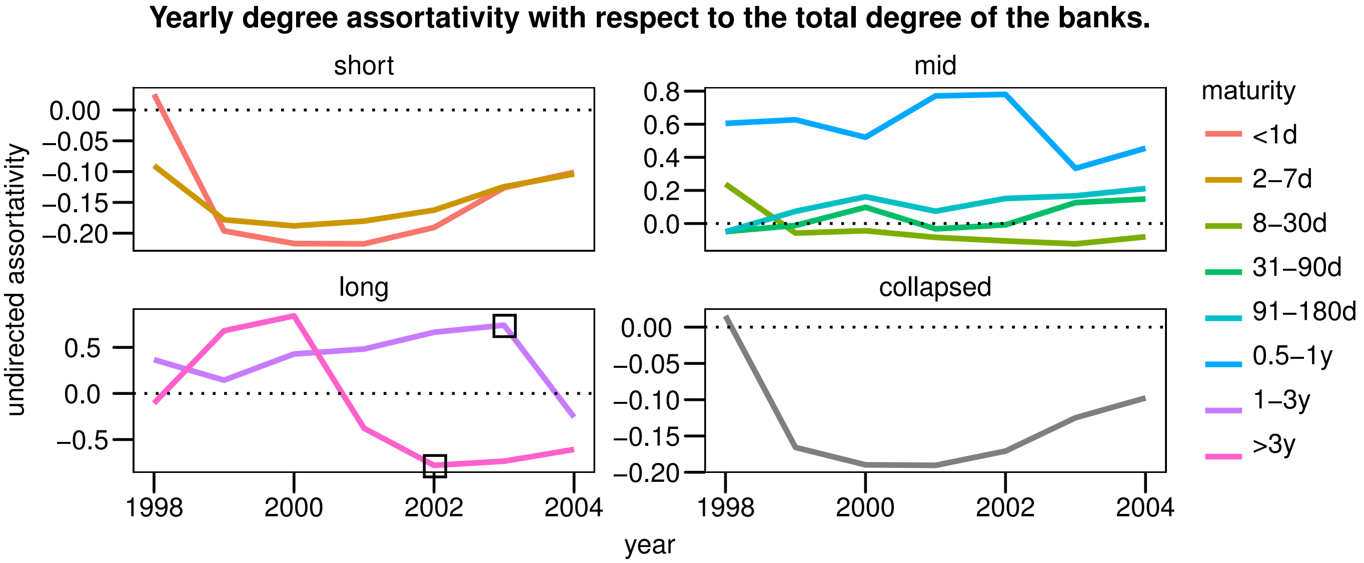

Figure 10. The degree assortativity measures to which extent nodes with a given degree associate preferentially with other nodes of similar degree – see Equation (2) in[17] for the formal definition. In this case the type of degree considered is the total degree, i.e. the sum of the in- and outdegree. A yearly resolution was chosen because monthly aggregation suffered from bad statistics starting from $G_{91-180d}$.

Summary

A summary of the results of this maturity layer analysis can be found in Table 1 in the main text. While certain features here are indicative of known structures in the interbank lending network literature, we refrain from making any such claims. Rather we

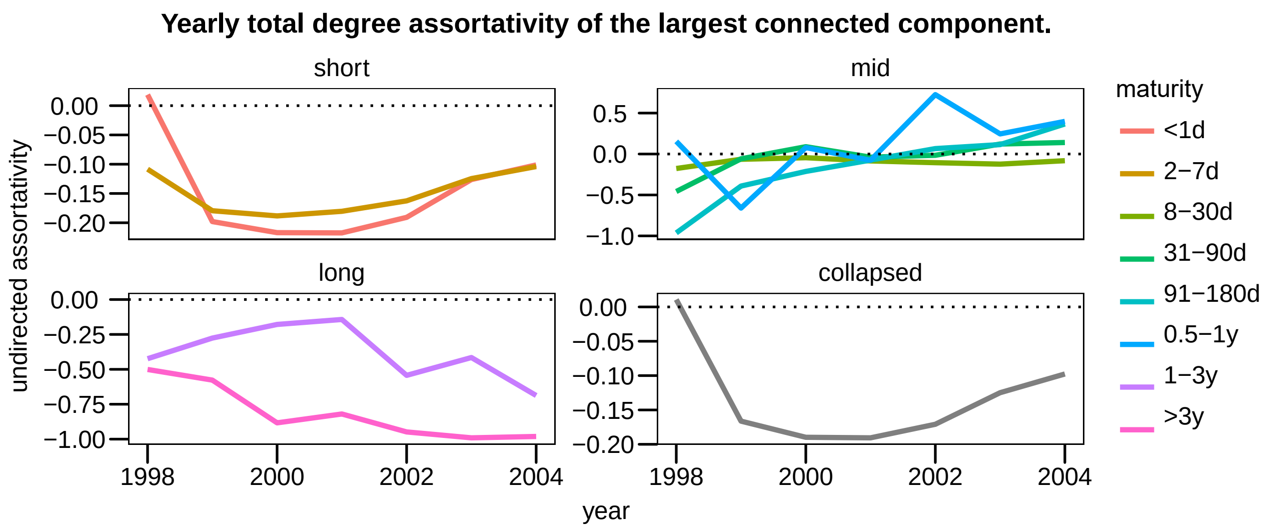

Figure 11. The degree assortativity for the total degrees throughout the years. The degree assortativity is also called degree correlation[8].

report the findings here as is and only make claims about function and structure in the main text where steps have been taken to gather statistical evidence for both the construction of the layers as the occurrence of structure in such layers.

B OG inference with non-contiguous binning

Here we present the results of the OG inference where non-contiguous binning was allowed in Fig. 12 (as opposed to the contiguous binning in the main text). This allows for partitions where short maturity loans are merged with long maturity loans. As explained in the main text, the creation of a new bin in the OG needs to be warranted by enough statistical evidence for it to be necessary; i.e. that it will contain a lending pattern that is significantly different from the lending patterns in the other bins. In those cases where very long maturity loans are merged with short maturity loans, we suspect that there is hardly any actual structure (and thus not enough statistical weight) in the long maturity layer due to its sparcity. It then makes sense that such layer is merged with the densest layer available since this merging will cause the least amount of new connections to be introduced relative to the other, less dense, bins.

Comparing Fig.3 (a) and (b) in the main text to Fig. 12 (a) and (b) shows the results to be qualitatively the same.

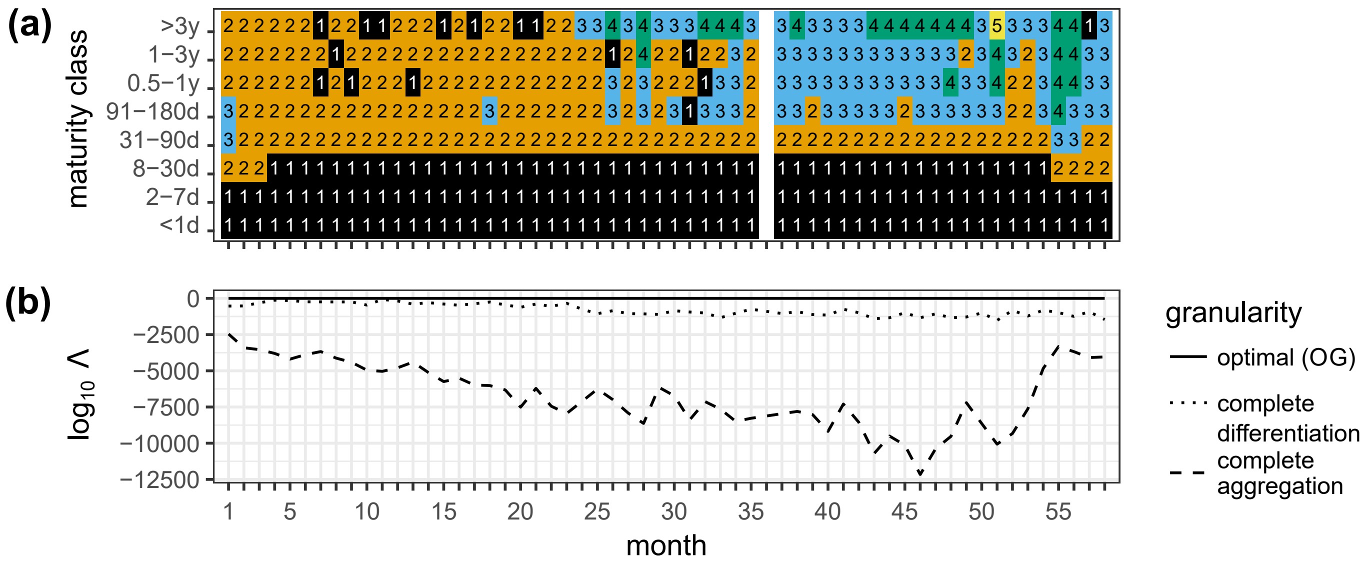

Figure 12. Results of the layered SBM with non-contiguous binning. The optimal granularity (OG) with respect to the loan maturity classes for the Russian interbank lending network. The OG corresponds to the granularity of the best-fit layered SBM. **(a)** Monthly time series of the OG inferred from the monthly interbank lending network. Each OG bin (OGB) holds one or more maturity classes and is labelled by an OGB index $1, 2, 3, 4$ and indicated by a colour. The OGB index runs according to the shortest maturity class in the bin. The bin with the shortest maturity class in it is numbered OGB 1, etc. The OGBs correspond to lending patterns between the bank groups that differ from each other in a statistically significant way. **(b)** Temporal evolution of the $\log_{10}$ of the posterior odds ratio Λ (for its definition see Eq. 4 in the main text) of the layered SBM for three different granularities: (i) the OG; (ii) complete loan maturity differentiation; (iii) complete loan maturity aggregation. Note that the OG inferred from the algorithm is consistently preferred. Complete aggregation is decisively rejected as an optimal representation of the mesoscale of the monthly interbank lending network

.

C Number of active banks per group

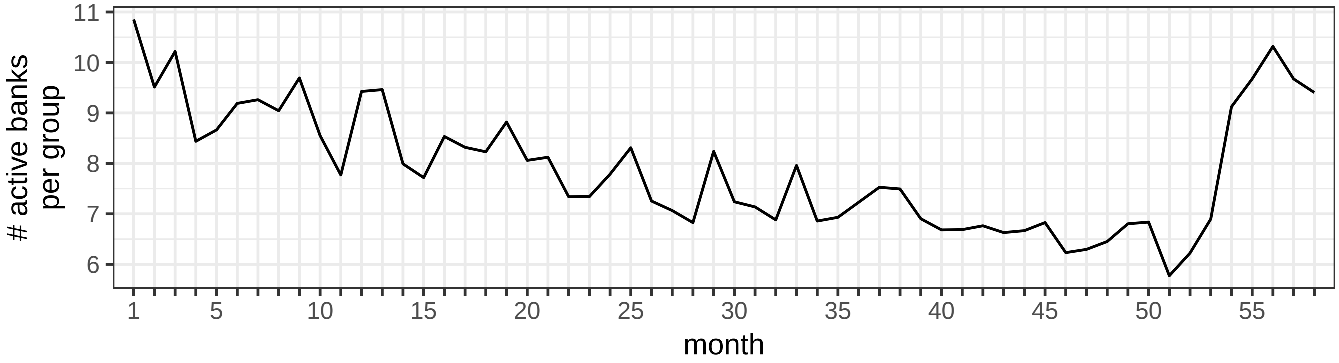

Figure 13. The number of active banks per group is obtained by dividing the number of active banks by B, i.e. the number of groups in the inferred bank groups $\{b_i\}$.

D Activity characteristics per OGB

We investigate the OGBs and their typical characteristics. Fig. 14 shows the distribution of the log group strengths across time and per OGB. We discern three types of group strength: outstrength, instrength and internal strength. The group outstrength in a certain month of bank group b is the total amount lent by all banks belonging to b to banks belonging to another group $b' \neq b$. In symbols,

$$s_b^{\text{out}} = \sum_l \sum_{i,j,k} x_{ijk}^l \delta_{b_i,b}(1 - \delta_{b_j,b}) \tag{7}$$

The two other strength types and the constraint that the maturity layer l must be in a given OGB are obtained likewise from this definition.

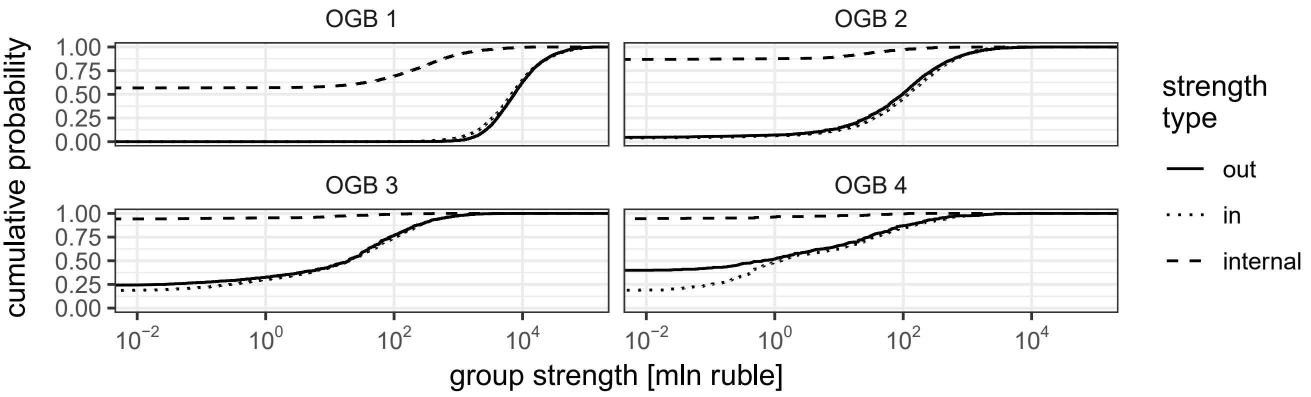

Figure 14. The time-integrated cumulative density functions (CDFs) of the group instrengths, outstrengths and internal strengths for each OGB. Zero group strengths are represented by non-vanishing CDFs at the origin.

We see that the groups for OGBs 2, 3, 4 show definite "anti-community characteristics", i.e. they hardly lend or borrow internally. There is also an interesting asymmetry between group instrength and group outstrength in OGB 4. Looking at the group strength modes, each OGB is seen to function on its own magnitude scale (see Fig. 5 in the main text).

E Other percentiles for instrength/strength of important banks

We look at the lending behaviour of individual banks. As in the main text we again use the bank strength to single out the "important" banks. We show that the conclusions from the main text hold for different bank strength cutoffs. Each row of subplots in Fig. 15 uses a different cutoff, respectively with the bank strength (s) unlimited, limited to the top 10%, top 5%, and the top 1%. At monthly time scales the important banks in "short" bins tend to both lend and borrow equal amounts of money, while the important banks in the "long" bins tend to either lend or borrow.

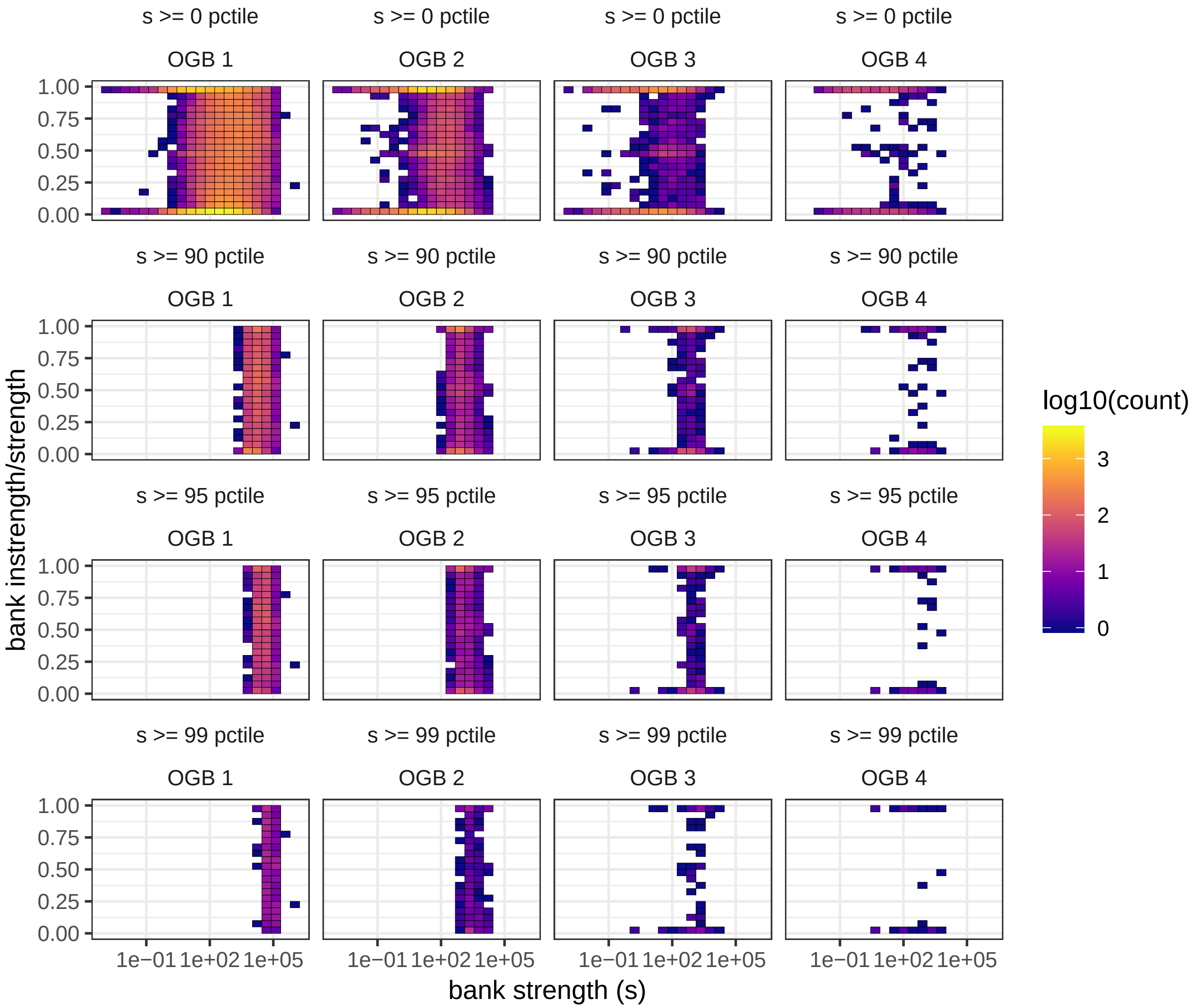

Figure 15. The time-integrated distribution of the strength and the instrength/strength ratio of the monthly "important banks" for the four OGBs. As in Fig. 4(b) in the main text we gauge a bank's importance by its strength: For a given month a bank is deemed important if its strength lies in the top X, with X varying per row of subplots (the top $X\%$ is defined by the $(1-X)$th percentile). With growing OGB index, the important banks increasingly tend to either lend or borrow. Banks in OGB 1 and OGB 2 tend to balance lending and borrowing. This suggests that the economic function of the important banks in the various OGBs changes from financial intermediation (short maturity bins) to financing (long maturity bins) on a monthly time scale.

F The trust crisis

The trust crises consist of the announcement crisis in the second half of 2003 and the summer of 2004 crisis[7, 18–20].

end of July 2003	Start of announcement crisis. Decline in reciprocal bank interactions.
September 2003	The network starts to fall apart because of the distrust among banks.
December 2003 - January 2004	Back to normal liquidity.
March	Peak of interbank lending market recovery from early trust crisis. The bank lending reciprocity, however, does not regain pre-crisis levels.
following weeks	Three stages of the summer of 2004 crisis.
First stage: April	Volatility on the interbank market with even higher lending rates than later stages but without significant outflow of individual's deposits. Demand for liquidity was caused by policy changes and statements of the CBR; no perception of crisis by the banks themselves, yet this financial instability undoubtedly impacted the crisis to come.
Second stage: May	CBR deprives Sodbusinessbank of its license.
May 19-21	Several conflicting statements from authorities about the deposit insurance for the clients of Sodbusinessbank make the depositors increasingly uneasy.
Third stage: June 3	Crisis now definitively developed beyond Sodbusinessbank alone. Banks start to introduce additional control measures; several suspend lending activities on the interbank market.
June 11	CBR changes policy rates to accommodate the banks, officially for the low liquidity on the interbank short-term market.
June 21-22	Peak of the crisis.
July 13	Interbank market starts to stabilize.
July 16	The 'crisis of confidence' is declared to be at an end.

Investigations of money laundering led the CBR to deprive Sodbusinessbank of its license in May 2004. The following mutual suspicion led to a drying up of liquidity on the interbank market in the summer of 2004. Roughly one year earlier, this investigation was announced, which caused a smaller trust crisis (i.e. the announcement crisis). In May 2004, a crisis on the Russian interbank market was triggered. In particular, on May 13 the Central Bank of Russia recalled the license from Sodbiznesbank on accusations of money laundering and sponsorship of terrorism. It was the first bank to be closed on these grounds in Russia. This unexpected closure caused panic on the interbank market since banks suspected other banks would follow suit, but they had no reliable information on who these banks might be.

On May 16 the head of Federal Financial Monitoring Service, Viktor Zubkov, announced publicly that his agency suspected

another ten banks of similar violations. Because this announcement was not accompanied by the actual list of suspected banks, it just fuelled the hysteria on the interbank market. Rumours about the identities of these ten banks started to spread rapidly. Soon several inconsistent blacklists were circulating in the banking community as bankers tried to guess which banks were officially suspected of money laundering. Anecdotal evidence suggests that banks were actively helping to spread the rumour by removing themselves from the list and adding competitors in an attempt to escape the carnage. By consequence, The union of all the blacklists circulating in the banking community expanded in a few days to include dozens of banks, including several market leaders.

In the presence of total uncertainty about the quality of their counterparties, banks began to reduce limits on each other, which reverberated into an acute liquidity drought on the interbank market[21]. The turnover volume on the interbank market dropped spectacularly. Later the original source of the panic, Viktor Zubkov, shockingly announced that "the Federal Financial Monitoring Service has no blacklist". The deputy Minister of Internal Affairs similarly announced that "the Interior Ministry has no such list. We have no plans to persecute any banks."For further descriptions (in Russian) of this episode in Russian history see[22,23]. Up till now it remains unclear whether an official list actually existed. Important for us is that the 2004 meltdown on the Russian interbank market was based on rumours and unrelated to shocks to the fundamentals of Russian banks or the Russian economy at large.

This non-fundamental and exogenous mutual trust crisis provides a great example of what structural changes occur during a shock to the non-fundamentals of the ILN. The advantage of the trust crisis compared to the 2007-2009 financial crisis is that, while the financial crisis was caused elsewhere and only indirectly found its way into Russia (via oil-prices, etc.), the trust crisis had its epicenter inside of Russia.

G Maturity classes in the ILN literature

An overview of the ILN literature using multilayer approaches and how their granularity compares to the current dataset.

Table 4. Classifications of loan maturity in terms of our maturity classes used by the Central Bank of Russia and several studies that have included multilayered ILNs.

<table>
<tr><th></th><th><1d</th><th>2-7d</th><th>8-30d</th><th>31-90d</th><th>91-180d</th><th>0.5-1y</th><th>1-3y</th><th>>3y</th></tr>
<tr><td>Central Bank of Russia[24]</td><td>instant</td><td colspan="2">short term</td><td colspan="5">long term</td></tr>
<tr><td>Bargigli et al. (2015)[2]</td><td>overnight</td><td colspan="5">short term</td><td colspan="2">long term</td></tr>
<tr><td>Aldasoro and Alves (2016)[16]</td><td colspan="6">short term</td><td colspan="2">long term</td></tr>
<tr><td>Montagna and Kok (2016)[25]</td><td colspan="4">short term</td><td colspan="4">long term</td></tr>
</table>

H Term structure of interest rates and loan volumes in the interbank loan market

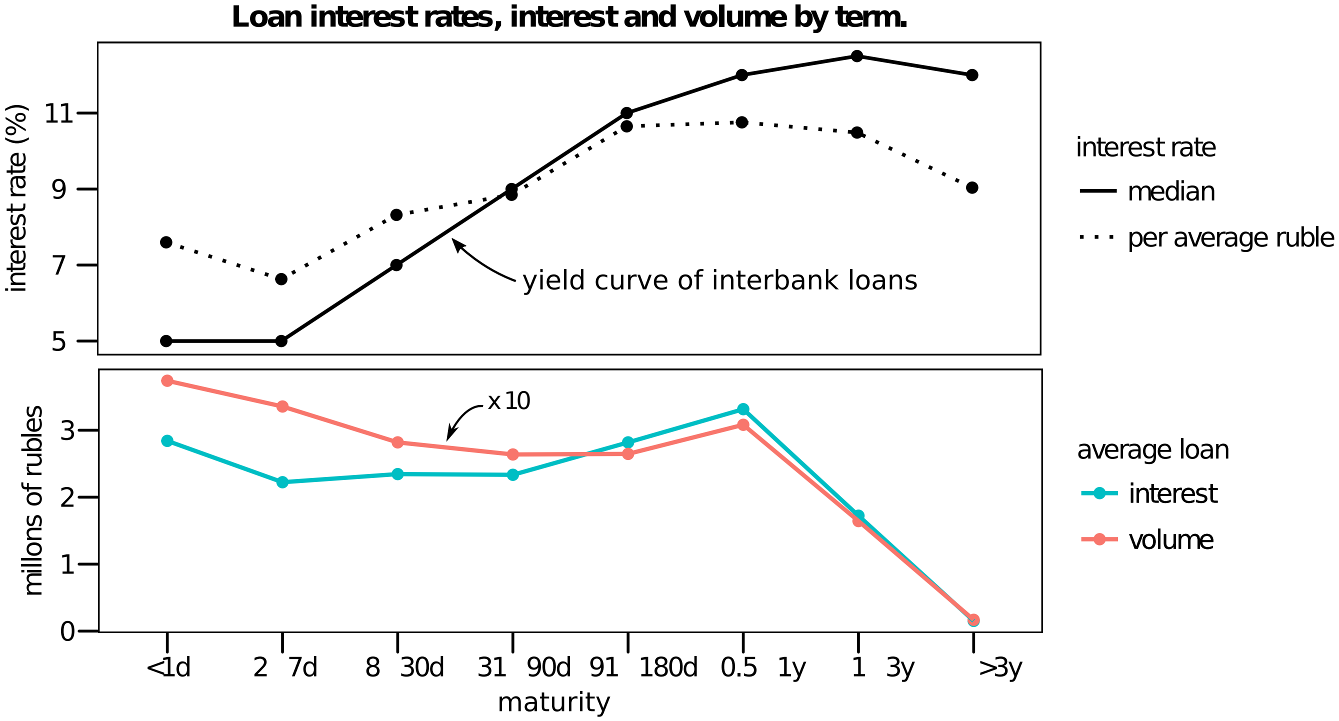

Figure 16. (top panel) The global categorical yield curve is the median interest rate over time per term. An alternative characterization is the interest rate per average ruble lent/borrowed, defined as the average interest weighed by the loan volumes. This statistic is robust to whether one excludes or includes the interest rate outliers. (lower panel) The average loan interest received/paid per term in millions of rubles and average loan volume (size) lent/borrowed in tens of millions of rubles.

The global categorical yield curve

The yield curve is obtained after "averaging" interest rates r over time and by maturity class. The averaging technique chosen is the median due to extreme outliers which disproportionately affect the mean: the standard deviation of the complete r population (including outliers) is $\sigma \approx 40\%$, yet 99% of the population is contained in $[0, \sigma]$. Within that interval the standard deviation is about 7%, which is a more sensible measure of the interest rate dispersion. By using the median, we avoid choosing any cutoff.

Yield curves are usually considered with *continuous* maturities ranging from one day to several years, but the available data only records maturity *classes*. Although methods exist to estimate the continuous yield curve from discrete data[26], we will only consider interest rates (and interests etc.) per category, i.e. per maturity class.

The solid line in the top panel of Figure 16 exhibits a typical stylized fact of the yield curve. It is upward sloping and has a convex shape, except for the >3y term, i.e. maturities three years or longer. The unusual steepness of the curve in Figure 16 is an artifact caused by the term categories; as they progress, they bucket a growing amount of maturities, so a continuous yield curve would be horizontally stretched with respect to the categorical yield curve. The upward slope is usually explained by classical *expectations theory* (ET). According to ET, interbank lending rates dynamics are determined by the structure

of liquidity supply and demand[27]; the long-term interest rate is an average of expected future short-term rates, plus a term premium that increases with longer terms to compensate risk-averse lenders for the interest risk, which arises for lenders from fluctuating interest rates with respect to the base deposit policy rate. One can also extend ET by including separate premiums for liquidity risk (selling loans on the secondary market tends to be harder as their maturities lengthen) and default risk, also called counterparty risk, which is in theory governed by the credit rating of the borrower. Normally the short-end (long-end) of the yield curve is dominated by the liquidity (default) risk; both are considered components of interbank lending risk[28,29]. Each risk contributes to the upward slope of the yield curve given normal market conditions.

The interest per average ruble is also displayed in the top panel of Figure 16. Loans with longer maturities are the most profitable for lenders. The drop for the >3y term could point to the fact that these contracts may be made on more amicable (flexible) terms; this would imply lower perceived interbank risk which could explain the negative slope between the 1-3y and >3y categories.

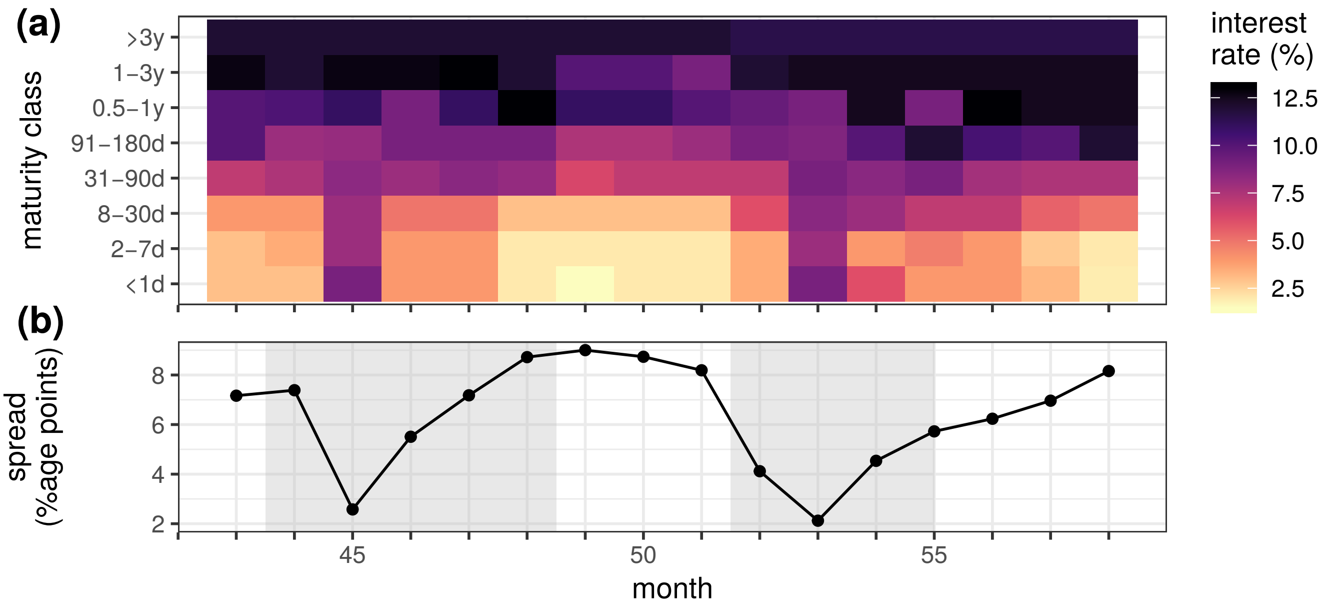

Figure 17. Monthly categorical yield curves (i.e. aggregated by maturity class; top panel) together with the spread (bottom panel) from July 2003 (month up until November 2004. This period is embedded in the emergent maturity phase of the network and includes the announcement and summer of 2004 crises (these are the trust crises – see Section F), both shaded in grey on the bottom panel. The first shaded region spans the period from August 1st 2003 until January 1st 2004. The second shaded region spans the April 1st 2004 until July 16th 2004. The CBR lowered the overnight rate from 16 to 14% on January 15, 2004 and again to 13% on June 15, 2004. The yield curve is the median interest rate per maturity class, as in Figures 16; the spread is defined in the text.

To illustrate the dynamics that underlie the global yield curve, Figure 17(a) plots the categorical yield curve for each of the last 16 months in the data. In this period the by now mature interbank network deals with the trust crises which are explained in more detail in Appendix F. Figure 17(b) contains the *(yield) spread*, which we define as the difference in average yield between "long" maturities and "short" maturities. We define these maturities as two classes: the "long maturity" class consists of maturity classes (1-3y, >3y) and the "short maturity" class consists of maturity classes (<1d, 2-7d, 8-30d). The general shape

of the curve is not sensitive to the definition of the "long maturity" class; the "short maturity" class is motivated in the paper, while the "long maturity class" is based on Basel recommendations and is also used in [2,16].

In normal times, interbank markets are among the most liquid in the financial sector: banks prefer to lend out excess cash since the central bank's interest rate on excess reserves is smaller than rates available in interbank markets[30]. During trust crises, the perceived default risk grows, which inflates interest rates according to ET. Riskier banks, i.e. banks at risk of being in financial distress, exert an externality on safer banks who subsidize their liquidity[30]. If the crisis gets worse, this externality on safer banks is so costly that they leave the unsecured market, and liquidity rich banks may prefer to hoard liquidity instead of lending it out to an adverse selection of borrowers; the interbank lending market dries up.

We see that this mechanism is indeed captured by the spread curve in the bottom panel of Figure 17: low spread seems to indicate abnormal market conditions with low liquidity because the short term interest rates increase quite relatively fast. Note in the upper panel that the long term interest rates stay almost constant during the crises and drop slightly during the recovery in between (roughly from January until April 2004). This suggests that we cannot make an analogy with the typical *inverted yield curves* of e.g. treasury securities that are associated with (predicting) recessions. Indeed, according to ET these are yield curves with negative slopes because investors have poor expectations of future interest rates. In contrast, we see for our data that the short term interest rates rise quickly during the trust crisis while the long term rates hardly change.

Characteristics of loan volumes and interests

The lower panel of Figure 16 shows the average interest and volume per term. The loan volumes are log-normally distributed[3] and this holds remarkably well for the interest too, especially for shorter terms. Because of the considerable variance on a linear scale, these averages may be interpreted only as rough order of magnitude estimates. With this in mind, Figure 16 shows that interest rate and volume are roughly negatively correlated: except for the bump at 0.5-1y and the case >3y, the volumes decrease as the interest rates increase. This can also be seen in the slowly varying average interest for the first five term categories.

Table 3 lists the total loan volumes by term and per year. As stated there (p. 3), we observe that the relative importance of each term segment, as measured by the total volume of loans traded within it, follows the ranking of the loan terms. This, together with the typical volumes and interest rates in Figure 16 and the lending activity in Table 2 (p. 3), supports the conclusion that the Russian interbank network exhibits a distinct hierarchy with respect to the loan maturities, which we could summarize by saying that *banks lend greater volumes at lower interest rates more often for shorter loan terms.*

References

1. Karas, A. & Schoors, K. Heracles or Sisyphus? Finding, cleaning and reconstructing a database of Russian banks. Working Papers of Faculty of Economics and Business Administration, Ghent University, Belgium 05/327, Ghent University, Faculty of Economics and Business Administration, https://ideas.repec.org/p/rug/rugwps/05-327.html (2005).

2. Bargigli, L., di Iasio, G., Infante, L., Lillo, F. & Pierobon, F. The multiplex structure of interbank networks. *Quant. Finance* **15**, 673–691 (2015).

3. Vandermarliere, B., Karas, A., Ryckebusch, J. & Schoors, K. Beyond the power law: Uncovering stylized facts in interbank networks. *Phys. A: Stat. Mech. its Appl.* **428**, 443–457 (2015).

4. Battiston, S., Caldarelli, G., May, R. M., Roukny, T. & Stiglitz, J. E. The price of complexity in financial networks. *Proc. Natl. Acad. Sci.* **113**, 10031–10036 (2016).

5. Battiston, F., Nicosia, V. & Latora, V. The new challenges of multiplex networks: Measures and models. *The Eur. Phys. J. Special Top.* **226**, 401–416 (2017).

6. Zlatic, V. *et al.* On the rich-club effect in dense and weighted networks. *The Eur. Phys. J. B* **67**, 271–275 (2009).

7. van den Heuvel, M. *Journey through the controllability of interbank credit networks*. mathesis, Ghent University, https://lib.ugent.be/catalog/rug01:002213899 (2015).

8. Newman, M. E. J. The Structure and Function of Complex Networks. *SIAM Rev.* **45**, 167–256 (2003).

9. Christensen, K. & Moloney, N. R. *Complexity and criticality*, vol. 1 (Imperial College Press, 2005).

10. Newman, M. E. J. Power laws, pareto distributions and zipf's law. *Contemp. Phys.* **46**, 323–351 (2005).

11. Watts, D. J. & Strogatz, S. H. Collective dynamics of small-world networks. *Nature* **393**, 440–442 (1998).

12. Silva, T. C., da Silva, M. S. & Tabak, B. M. Liquidity Performance Evaluation of the Brazilian Interbank Market using a Network-Based Approach. Working Papers Series 401, Central Bank of Brazil, Research Department, https://ideas.repec.org/p/bcb/wpaper/401.html (2015).

13. Blasques, F., Bräuning, F. & Lelyveld, I. v. A dynamic network model of the unsecured interbank lending market. *J. Econ. Dyn. Control.* **90**, 310–342 (2018).

14. Vandermarliere, B. *Network analysis of the Russian interbank system*. mathesis, Ghent University, http://lib.ugent.be/catalog/rug01:001892466 (2012).

15. Craig, B. & von Peter, G. Interbank tiering and money center banks. *J. Financial Intermediation* **23**, 322–347 (2014).

16. Aldasoro, I. & Alves, I. Multiplex interbank networks and systemic importance: An application to European data. *J. Financial Stab.* **35**, 17–37 (2018).

17. Newman, M. E. J. Mixing patterns in networks. *Phys. Rev. E* **67**, 026126+ (2003).

18. Karas, A., Schoors, K. *et al.* Bank networks, interbank liquidity runs and the identification of banks that are too interconnected to fail. *Second. CInST Bank. Work. Mosc.* (2012).

19. Porfiriev, B. *Crises in Russia: Contemporary Management Policy and Practice From A Historical Perspective* (Routledge, New York, NY, USA, 2012).

20. van de Wiel, I. The 1998 Russian Crisis. Tech. Rep., Rabobank, https://economics.rabobank.com/publications/2013/september/the-russian-crisis-1998/ (2013).

21. Paranyushkin, D. Russian Money Market & Bank System: Problems (2009).

22. Simonov, N. S. *Banks and Money, 1988-2008* (RAGS, 2009).

23. Boyarskiy, A. Modern Russian Banking System. Brief Chronology. Period 1988-2004. (2018).

24. Guleva, V. Y., Skvorcova, M. V. & Boukhanovsky, A. V. Using multiplex networks for banking systems dynamics modelling. *Procedia Comput. Sci.* **66**, 257–266 (2015).

25. Montagna, M. & Kok, C. Multi-layered interbank model for assessing systemic risk. ECB Working Papers 1944, European Central Bank, https://ssrn.com/abstract=2830546 (2016).

26. Anatolyev, S. & Korepanov, S. The term structure of russian interest rates. *Appl. Econ. Lett.* **10**, 867–870 (2003).

27. Egorov, A. & Kovalenko, O. Structural features and interest-rate dynamics of Russia's interbank lending market. BOFIT Discussion Papers 23/2013, Bank of Finland, Institute for Economies in Transition, https://ideas.repec.org/p/bof/bofitp/2013_023.html (2013).

28. Tabak, B. & Andrade, S. Testing the expectations hypothesis in the brazilian term structure of interest rates. *Braz. Rev. Finance* **1**, 19–43 (2003).

29. Russell, S. *et al.* Understanding the term structure of interest rates: the expectations theory. *Fed. Reserv. Bank St. Louis Rev.* **74**, 36–50 (1992).

30. Heider, F., Hoerova, M. & Holthausen, C. Liquidity hoarding and interbank market rates: The role of counterparty risk. *J. Financial Econ.* **118**, 336 – 354 (2015).
